# Avian Metapneumovirus Subtype B at the Wildlife–Poultry Interface in Egypt: Molecular and Serological Insights into Cross-Ecological Transmission

**DOI:** 10.3390/v18060591

**Published:** 2026-05-24

**Authors:** Omar S. Saeed, Sara A. Shabana, Mahmoud Gamal, Basem M. Ahmed, Ayman H. El-Deeb, Haitham M. Amer

**Affiliations:** 1 Department of Virology, Faculty of Veterinary Medicine, Cairo University, Giza 12211, Egypt; basem-ahmed@cu.edu.eg (B.M.A.); ayman_vv@cu.edu.eg (A.H.E.-D.); hamoamer@cu.edu.eg (H.M.A.); 2 Independent Researcher, Giza 12211, Egypt; vet.sara.alaa@gmail.com; 3 Department of Biochemistry and Molecular Biology, Faculty of Veterinary Medicine, Cairo University, Giza 12211, Egypt; mahmoud.gamal@cu.edu.eg; 4 Center for Biotechnology and Interdisciplinary Studies, Rensselaer Polytechnic Institute, Troy, NY 12180, USA; 5 Faculty of Veterinary Medicine, King Salman International University, El-Tor 46618, Egypt; 6 Faculty of Veterinary Medicine, Egyptian Chinese University, Cairo 11437, Egypt

**Keywords:** aMPV, molecular epidemiology, serological surveillance, poultry interface, wild birds, RT-qPCR

## Abstract

Avian metapneumovirus (aMPV) is a major respiratory pathogen of poultry with a significant economic impact; however, its epidemiology at the wildlife–poultry interface remains poorly understood, particularly within Afro–Eurasian migratory systems. This cross-sectional study (December 2024–April 2026) investigated aMPV occurrence in wild birds across eleven Egyptian governorates representing key ecological zones along major migratory flyways. A total of 1280 samples were collected from 800 wild birds representing migratory waterfowl and synanthropic species, including 800 oropharyngeal swabs tested by real-time RT-qPCR for aMPV subtypes A and B and 480 serum samples analyzed using indirect ELISA. aMPV RNA was detected in 28/800 samples (3.5%), with all positives identified as subtype B and confined to the Nile Delta, Middle Egypt, and Canal Region. In contrast, serological analysis revealed a high seroprevalence of 58.3% (280/480), indicating widespread prior exposure with significant spatial and species-level variation (*p* < 0.05). The marked disparity between low molecular detection and high seroprevalence supports transient infection with cumulative exposure. The exclusive detection of subtype B may reflect epidemiological connectivity between poultry and wild bird populations within shared ecological interfaces; however, the directionality of transmission and the possibility of independent wildlife maintenance could not be determined within the scope of the present cross-sectional study. Future studies incorporating whole-genome sequencing, longitudinal surveillance, and broader flyway-scale sampling are needed to resolve transmission pathways and distinguish field strains from potential vaccine-derived viruses within wildlife–poultry interfaces.

## 1. Introduction

Avian metapneumovirus (aMPV), historically referred to as avian pneumovirus (APV) or turkey rhinotracheitis (TRT) virus, is a globally important respiratory pathogen of poultry that continues to impose substantial economic constraints on commercial production systems. Clinically, infection is characterized by upper respiratory tract disease, including sinusitis, nasal and ocular discharge, facial oedema, swollen head syndrome (SHS), reduced egg production, and increased mortality, particularly in turkeys and chickens. In addition to direct clinical impacts, aMPV contributes significantly to production losses by predisposing infected flocks to secondary bacterial infections, most notably *Escherichia coli*, *Mycoplasma gallisepticum*, and *Ornithobacterium rhinotracheale*, thereby exacerbating disease severity and complicating diagnosis and control strategies [1,2,3].

Taxonomically, aMPV belongs to the genus *Metapneumovirus* within the family *Pneumoviridae*. It is an enveloped, non-segmented, single-stranded negative-sense RNA virus that encodes eight structural proteins organized as N, P, M, F, M2, SH, G, and L genes. Among these, the attachment (G) glycoprotein exhibits the highest genetic variability and serves as the primary determinant for molecular subtyping, whereas conserved genes such as the nucleoprotein (N) are commonly targeted in molecular and serological diagnostic assays. Genetic and antigenic diversity underpin the classification of aMPV into four principal subtypes (A–D), with additional related lineages reported in avian metapneumoviruses worldwide. Subtypes A and B are widely distributed across Africa, Europe, Asia, and the Americas, subtype C is mainly restricted to North America, and subtype D has been reported only in limited historical isolations in Europe [4,5,6].

aMPV has been increasingly detected in wild bird populations across multiple continents, highlighting its broader ecological distribution beyond domestic poultry. Molecular surveys in Europe, particularly in Italy, have demonstrated the circulation of multiple aMPV subtypes (A, B, C, and novel variants) among free-ranging avifauna, including species such as *Anas platyrhynchos* (mallard), *Anas crecca* (Eurasian teal), *Fulica atra* (Eurasian coot), and *Larus michahellis* (yellow-legged gull), albeit at generally low prevalence levels. Notably, subtype C has been identified in migratory waterfowl such as *Mareca penelope* (Eurasian wigeon) in northeastern Italy, supporting the hypothesis that long-distance migratory species may facilitate viral dissemination across geographic regions [7,8,9].

Similar findings have been reported in North America, including Canada and the United States, where the sporadic detection of subtype C in wild waterfowl such as *Anas discors* (blue-winged teal) and *Branta canadensis* (Canada goose) indicates limited but widespread viral circulation [10].

Globally, accumulating evidence suggests that wild birds are more likely to function as incidental or bridging hosts rather than primary reservoirs of aMPV. A systematic review and meta-analysis confirmed that while serological evidence of exposure can be relatively common, active viral detection rates remain low in most wild bird populations [11]. However, the emergence of novel variants in Asia, particularly in China, associated with clinical disease in domestic and semi-domestic waterfowl such as *Tadorna tadorna* (common shelduck) and *Anas platyrhynchos domesticus* (domestic duck), underscores the potential for cross-species transmission and viral adaptation at the wildlife–poultry interface [12,13].

In contrast, their role is more consistently interpreted within a framework of ecological connectivity, particularly at wetland interfaces and along migratory flyways linking regions such as Europe, the Mediterranean basin, and Africa [11].

In Egypt, aMPV has been reported for several years as an important respiratory pathogen affecting commercial poultry production. Early studies linked aMPV infection with swollen head syndrome (SHS) and respiratory disease outbreaks in broiler chickens, which were associated with economic losses due to poor growth, reduced production performance, and secondary bacterial infections [14,15,16]. Subsequent serological investigations demonstrated widespread exposure to aMPV among commercial chicken and duck flocks in different Egyptian governorates, suggesting that the virus is widely distributed within the poultry sector [17].

Molecular studies later confirmed the circulation of different aMPV strains in Egypt, including both field and vaccine-related viruses [18,19]. More recently, serological and molecular investigations in unvaccinated broiler breeder flocks demonstrated widespread antibody detection together with active molecular circulation of aMPV, particularly subtype B, indicating ongoing field transmission under non-vaccinated conditions [20]. Collectively, these findings suggest that aMPV is continuously circulating within Egyptian poultry production systems.

Egypt possesses one of the largest and most densely interconnected poultry production sectors in the Middle East and North Africa, encompassing intensive commercial farms, semi-closed production systems, live bird markets, and extensive backyard poultry holdings that frequently coexist within the same geographic regions. Commercial broiler, breeder, layer, and duck farms are heavily concentrated in the Nile Delta and Canal governorates, particularly Dakahlia, Sharqia, Kafr El-Sheikh, Beheira, Gharbia, Ismailia, Port Said, and Damietta, where high poultry densities overlap with wetlands, irrigation networks, fish-farming areas, and major migratory bird habitats including Lake Manzala, Lake Burullus, and the Suez Canal corridor [21,22,23].

In parallel, rural poultry production remains an important component of traditional agriculture throughout the Nile Valley and Middle Egyptian governorates such as Fayoum, Beni-Suef, Minya, Assiut, and Sohag. Backyard flocks are commonly maintained under low-biosecurity scavenging systems in which chickens, ducks, geese, pigeons, and turkeys are raised in close proximity to human dwellings, cultivated land, irrigation canals, and free-ranging synanthropic birds. Informal poultry holdings are also frequently established near drainage canals, fishponds, and peri-wetland environments, increasing opportunities for indirect interaction between domestic poultry and migratory waterfowl [24,25,26].

The Egyptian poultry industry operates through a vertically interconnected production structure involving breeder farms, hatcheries, broiler and layer operations, duck farms, and live bird markets linked through continuous poultry movement and trade. Although modern environmentally controlled housing systems are increasingly being adopted in large commercial farms, traditional open-sided houses and backyard production systems remain widespread, particularly in rural and peri-urban areas [27].

The implementation of Biosecurity across Egyptian poultry sectors remains variable and strongly associated with farm scale and management capacity. Small-scale commercial farms and backyard systems frequently exhibit inadequate biosecurity measures, including limited sanitation infrastructure, poor visitor control, insufficient carcass disposal practices, and weak separation between poultry species and production sectors. These conditions may facilitate viral circulation and persistence in densely populated poultry regions [28,29].

Such ecological and production characteristics have historically supported the endemic circulation of several avian pathogens in Egypt, including highly pathogenic avian influenza viruses and infectious bursal disease virus, despite ongoing vaccination programs and control efforts [30]. The close coexistence of intensive poultry production, backyard flocks, wetlands, and migratory bird habitats creates favorable conditions for pathogen maintenance and potential interspecies transmission at the wildlife–poultry interface.

Within this context, Egypt represents a critical yet underexplored epidemiological setting. Positioned at the intersection of the Black Sea–Mediterranean and East Africa–West Asia migratory flyways, the country serves as a major stopover and wintering site for migratory waterbirds. These flyways converge at key ecological hotspots, particularly the Nile Delta and Suez region, where high densities of wild birds overlap with intensive poultry production systems [22,23]. This ecological interface may facilitate viral exchange between wild and domestic avian populations, highlighting the importance of Egypt in understanding aMPV transmission dynamics.

From a diagnostic perspective, virus isolation is often limited by short viral shedding periods and sample degradation. In experimentally infected chickens, aMPV subtype B shedding typically occurs between 3 and 7 days post-infection, peaking around day 5, which restricts the window for successful detection [31,32,33]. In contrast, reverse transcription real-time PCR (RT-qPCR) provides rapid, sensitive detection and is considered the preferred method for routine surveillance and subtype identification [34]. Serological assays, particularly ELISA targeting the conserved N protein, are widely used for population-level surveillance, enabling detection of past exposure and assessment of infection pressure despite not distinguishing subtypes [35].

Accordingly, the present study was designed to investigate the molecular occurrence and seroprevalence of aMPV in wild and migratory birds across key ecological zones in Egypt during 2024–2026. By integrating molecular and serological approaches within a flyway-informed ecological framework, this study provides the first comprehensive assessment of aMPV at the wildlife–poultry interface in Egypt and contributes to a refined understanding of its epidemiological dynamics in a transboundary context.

## 2. Materials and Methods

### 2.1. Study Area and Ecological Stratification

Sampling was conducted across eleven Egyptian governorates selected to represent key ecological interfaces along major migratory flyways and intensive poultry production systems. The study area was stratified into four ecological zones reflecting gradients in poultry density, wetland distribution, and migratory bird activity: the Nile Delta (Sharkia, Dakahlia, Kafr El-Sheikh, Beheira, Gharbia), the Canal Region (Port Said, Ismailia), Middle Egypt (Fayoum, Giza), and Upper Egypt (Minya, Assiut).

Sampling was ecologically weighted rather than equally distributed, with proportional representation based on field accessibility and ecological relevance. Most samples originated from the Nile Delta (57.9%), followed by Middle Egypt (20.5%), Upper Egypt (12.6%), and the Canal Region (9%), reflecting regional differences in poultry density and wetland-associated bird abundance.

Target birds were categorized according to their ecological and epidemiological relevance. Migratory and wetland-associated waterbirds represented the largest group and included *Spatula clypeata*, *Mareca penelope*, *Anas crecca*, *Fulica atra*, and *Larus ridibundus*. Synanthropic and peri-domestic species frequently associated with poultry production environments included *Passer domesticus*, *Columba livia*, *Columba oenas*, *Corvus cornix*, and *Bubulcus ibis*. Additional sampled species included *Coturnix coturnix*, a ground-dwelling migratory granivorous bird commonly encountered in agricultural habitats near poultry production systems.

### 2.2. Sample Collection and Processing

A total of 1280 samples were collected from 800 wild birds during two consecutive migratory surveillance periods extending from December 2024 to April 2025 and from October 2025 to April 2026, corresponding to the major migratory and overwintering periods in Egypt. Sampling was conducted intermittently within these defined seasonal windows rather than continuously throughout the study period. Surveillance activities targeted poultry–wild bird interface areas, including wetlands, irrigation canals, agricultural fields, and water bodies located near poultry production environments. Birds were captured using mist nets or through live capture by local hunters operating within the surveillance areas. Following capture, the birds were carefully restrained and examined by trained veterinarians to minimize handling-related stress and injury [36]. Oropharyngeal swabs and blood samples were immediately collected after their capture under field biosafety conditions. After sampling, the birds were briefly monitored and released at the site of capture whenever their condition permitted safe release.

**Figure 1:**
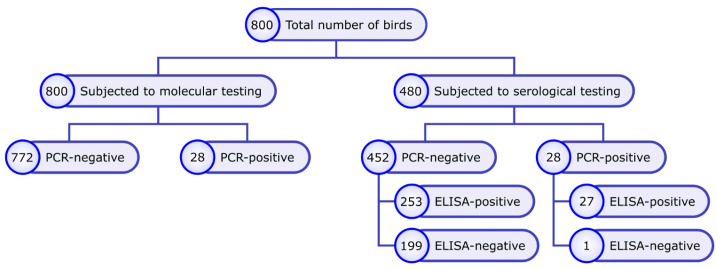
Study design and sampling framework for molecular and serological screening of wild birds. A cross-sectional study design was implemented to investigate avian metapneumovirus (aMPV) circulation in wild birds. A total of 800 wild birds were included and subjected to molecular screening using real-time RT-qPCR for aMPV detection. From the same study population, 480 individuals were further selected for serological investigation using an indirect ELISA to assess exposure history. The sampling strategy was structured to enable parallel molecular detection of viral RNA and serological assessment of aMPV-specific antibodies, allowing integrated analysis of infection dynamics at the wild bird population level.

Seasonal timing within each migratory period was not incorporated as an independent analytical variable because the study was primarily designed as an interface-based surveillance rather than a temporal ecological assessment. Owing to field sampling constraints, species-specific handling considerations, and variable sample quality under field conditions, not all birds yielded both sample types. Consequently, the final dataset included 800 oropharyngeal swab samples for molecular analysis and 480 serum samples for serological testing after quality assessment and exclusion of unsuitable or insufficient specimens as shown in Figure 1.

Oropharyngeal swabs were aseptically collected using sterile polyester-tipped applicators and immediately immersed in 3 mL of viral transport medium (VTM). The VTM consisted of modified Hanks’ Balanced Salt Solution (HBSS; Thermo Fisher Scientific, Waltham, MA, USA) supplemented with 2% heat-inactivated fetal bovine serum (FBS; Gibco, Thermo Fisher Scientific, Grand Island, NY, USA)gentamicin (100 µg/mL; Sigma-Aldrich, St. Louis, MO, USA) and amphotericin B (0.5 µg/mL; Sigma-Aldrich, St. Louis, MO, USA) to ensure microbial suppression. Samples were transported under refrigerated conditions (2–8 °C) in insulated containers and processed promptly upon arrival at the Virology Laboratory, Faculty of Veterinary Medicine, Cairo University. Swab suspensions were vortexed, briefly clarified by centrifugation, aliquoted to avoid repeated freeze–thaw cycles, and stored at −80 °C until molecular analysis.

For serological investigations, approximately 1.5 mL of blood was aseptically collected from the wing vein of each bird into sterile tubes. Following clot formation at room temperature, samples were centrifuged at 1006× *g* for 10 min. The separated sera were transferred into labeled microtubes, documenting sample metadata (location, species, and collection date), and stored at −20 °C until testing. Samples exhibiting hemolysis and/or contamination were excluded from further analysis.

### 2.3. RNA Extraction and Molecular Detection by RT-qPCR

Viral RNA was extracted from 200 µL of individual oropharyngeal swab material using the QIAamp Viral RNA Mini Kit (Qiagen, Hilden, Germany), following the manufacturer’s instructions under RNase-free conditions. Extracted RNA was eluted in nuclease-free buffer and stored at −80 °C until amplification.

Avian metapneumovirus (aMPV) subtypes A and B were detected using the Kylt^®^ aMPV A/B real-time RT-PCR kit (AniCon Labor GmbH, Emstek， Germany) on an ABI 7500 Fast Real-Time PCR system (Thermo Fisher Scientific, Waltham, MA, USA). The assay targets the viral G gene using subtype-specific TaqMan probes labeled with FAM (aMPV-A) and Cy5 (aMPV-B) whereas β-actin was included as an endogenous internal control (HEX channel) to validate RNA integrity and reaction performance [34].

Each reaction consisted of 16 µL of master mix and 4 µL of RNA template. Thermal cycling conditions included reverse transcription at 50 °C for 10 min, initial denaturation at 95 °C for 1 min, followed by 42 amplification cycles comprising denaturation at 95 °C for 10 s and annealing/extension at 60 °C for 60 s with fluorescence acquisition.

Positive controls included commercially available live attenuated vaccines for aMPV subtype A (Poulvac^®^ TRT, Zoetis, Kalamazoo, MI, USA) and subtype B (Nemovac^®^, Merial, Lyon, France), while nuclease-free water was used as a negative control. Samples with cycle threshold (Ct) values ≥ 37 were considered negative. Amplification profiles showing a difference greater than 10 cycles between the subtype-specific channels were interpreted as inconclusive.

### 2.4. Serological Analysis

#### 2.4.1. Indirect ELISA Screening

Serum samples were analyzed for the presence of antibodies against aMPV subtypes A and B using a commercial indirect ELISA kit (IDvet^®^, Grabels, France), according to the manufacturer’s protocol. Although the assay was originally developed and validated for domestic poultry, no commercially validated serological assays are currently available for most migratory wild bird species. Therefore, the assay was used as an exploratory serological screening tool for wildlife surveillance purposes rather than as a definitive confirmatory diagnostic assay. Briefly, sera were diluted to 1:500 and added to antigen-coated microtiter plates. Following incubation and washing steps, horseradish peroxidase (HRP)-conjugated anti-chicken antibodies were applied, and color development was achieved using tetramethylbenzidine (TMB) as the substrate.

Optical density (OD) values were measured at 450 nm using a microplate reader (Multiskan FC™, Thermo Fisher Scientific, Waltham, MA, USA). Each plate included duplicate wells of positive and negative controls provided by the manufacturer to ensure assay validity. The assay was considered valid when the mean OD of the positive control exceeded 0.250 and the ratio of positive to negative control OD values was greater than 3. The sample-to-positive (S/P) ratio was calculated using the following formula:

(Sample OD—Negative Control OD)/(Positive Control OD—Negative Control OD).

Antibody titers were subsequently derived using the equation:

Log_10_(Titer) = 1.09 × Log_10_(S/P) + 3.360.

Because the ELISA kit was originally validated for chickens, the suitability of the manufacturer-recommended cutoff (titer > 396) was reassessed for the investigated wild bird species. An alternative cutoff was empirically estimated from the ELISA titer distribution using kernel density estimation, with the threshold defined at the local minimum separating the major density peaks. The derived cutoff (titer > 613) was approximately 1.5-fold higher than the manufacturer-recommended threshold; however, its application altered the estimated seroprevalence by only 0.2%, corresponding to the reclassification of a single bird. ELISA titer distribution and cutoff estimations are presented in Supplementary Figure S1.

#### 2.4.2. Antigen Inhibition Assay for Assessment of ELISA Specificity

To further assess the specificity of positive ELISA reactions detected in wild-bird sera, a quantitative inhibition assay was performed using three sera positive for both RT-qPCR and indirect ELISA.

A live attenuated aMPV subtype B vaccine (Nemovac^®^, Merial, Lyon, France) was used as a competing antigen source. The vaccine antigen was reconstituted according to the manufacturer’s instructions and serially diluted (1:2 to 1:32) in phosphate-buffered saline (PBS). Equal volumes of diluted serum and diluted vaccine antigen (100 µL each) were mixed and incubated for 1 h at 37 °C prior to retesting using a commercial indirect ELISA kit (IDvet^®^, Grabels, France). Uninhibited serum incubated with PBS served as the control reaction. Optical density (OD) values were measured at 450 nm, and the percentage inhibition (PI) was calculated as follows:

PI (%) = [(ODcontrol − ODinhibited)/ODcontrol] × 100

where ODcontrol represents the OD value of uninhibited serum and ODinhibited represents the OD value following pre-incubation with the vaccine antigen. This supplementary assay was performed as an exploratory specificity assessment and was not included in the routine diagnostic workflow.

### 2.5. Statistical Analyses

Serological data were compared across regions, governorates, and species using the Kruskal–Wallis test, followed by pairwise post hoc comparisons using Dunn’s test with *p*-value adjustment according to the Benjamini–Hochberg procedure. Spearman’s rank correlation analysis was performed to assess the relationship between PCR-positive Ct values and antibody titers. All statistical analyses and data visualizations were conducted in RStudio, Boston, MA, USA (version 2025.09.2) [37] using the R environment, Vienna, Austria (version 4.5.2) [38]. Data are presented as boxplots with individual data points, and statistical significance was set at *p* < 0.05.

## 3. Results

### 3.1. Molecular Detection of aMPV in Wild Birds (RT-qPCR)

aMPV RNA was detected in 28 of 800 oropharyngeal swab samples from wild bird populations sampled across four Egyptian regions, corresponding to a molecular detection rate of 3.5%. All positive samples were confirmed to be subtype B, while subtype A was not detected in any of the analyzed specimens. Positive detections were confined to the Nile Delta, Middle Egypt, and the Canal Region, whereas no aMPV RNA was detected in samples collected from Upper Egypt. A comprehensive breakdown of the RT-qPCR and ELISA results across governorates, species, and ecological groups is provided in Supplementary Table S1.

In the Nile Delta, molecular positivity was detected across several governorates and species. In Beheira, aMPV RNA was identified in *Passer domesticus* (3/40; 7.5%), *Spatula clypeata* (6/55; 10.9%), and *Mareca penelope* (2/30; 6.67%), while *Anas crecca* tested negative (0/10; 0%). In Kafr El-Sheikh, positive detections were recorded in *Spatula clypeata* (5/72; 6.9%), *Mareca penelope* (1/4; 25%), and *Fulica atra* (1/80; 1.25%). In Sharkia, both *Mareca penelope* and *Columba livia* showed identical detection rates (1/20; 5% each). In Dakahlia, *Fulica atra* (1/40; 2.5%) and *Coturnix coturnix* (1/30; 3.3%) were positive, while in Gharbia, *Passer domesticus* showed a detection rate of 1.6% (1/62) as shown in Table 1.

Middle Egypt, no aMPV RNA was detected in *Bubulcus ibis* from Fayoum (0/92; 0%). However, in Giza, aMPV RNA was detected in *Passer domesticus* (1/20; 5%) and *Bubulcus ibis* (1/52; 1.9%). In the Canal Region, positive detections were recorded in *Larus ridibundus* from Ismailia (2/30; 6.7%) and *Corvus cornix* from Port Said (1/42; 2.4%). No molecular positivity was detected in Upper Egypt, where all samples from Minya (*Columba oenas*, 0/61) and Assiut (*Corvus cornix*, 0/40) were negative.

### 3.2. Seromonitoring

#### 3.2.1. Serological Evidence of aMPV Exposure

A total of 480 serum samples collected from wild birds representing multiple species and geographic locations across Egypt were analyzed for antibodies against avian metapneumovirus (aMPV). Overall, 280 samples tested positive, yielding a seroprevalence of 58.3%. Seropositive birds were detected in the Nile Delta, Middle Egypt, and the Canal Region, whereas no seropositivity was recorded in Upper Egypt. In the Nile Delta, a consistently high seroprevalence was observed across multiple governorates and species.

In Beheira, antibodies were detected in *Passer domesticus* (13/20; 65%), *Spatula clypeata* (44/55; 80%), *Mareca penelope* (15/15; 100%), and *Anas crecca* (8/10; 80%). In Kafr El-Sheikh, the seroprevalence reached 96.67% (29/30) in *Spatula clypeata*, 100% (4/4) in *Mareca penelope*, and 11.1% (2/18) in *Fulica atra*. In Sharkia, antibodies were detected in *Columba livia* (18/20; 90%) and *Mareca penelope* (4/15; 26.6%). In Dakahlia, *Fulica atra* and *Coturnix coturnix* showed seroprevalence values of 80% (16/20) and 84% (21/25), respectively, while in Gharbia, *Passer domesticus* demonstrated a seroprevalence of 75% (30/40).

In Middle Egypt, high seropositivity was recorded in *Bubulcus ibis* from Fayoum (37/43; 86%), while in Giza, *Passer domesticus* and *Bubulcus ibis* showed seroprevalence values of 75% (15/20) and 61% (11/18), respectively. In the Canal Region, seropositivity was detected in *Larus ridibundus* from Ismailia (13/25; 52%), whereas *Corvus cornix* from Port Said showed no detectable antibodies (0/30; 0%). In Upper Egypt, all tested birds were seronegative, including *Columba oenas* from Minya (0/39) and *Corvus cornix* from Assiut (0/33).

**Figure 2:**
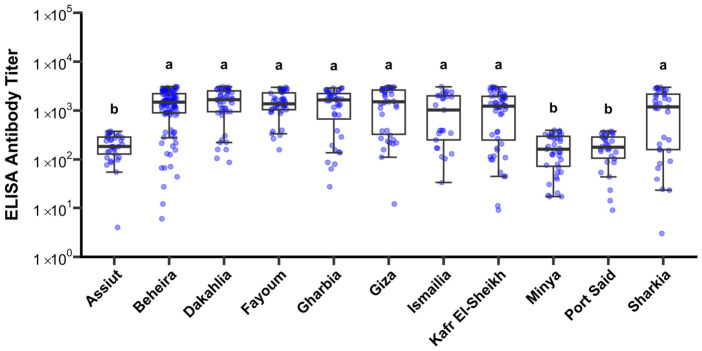
Governorate-specific ELISA antibody titers against avian metapneumovirus (aMPV) detected in wild birds sampled across Egypt during 2024–2026. Differences among governorates were evaluated using the Kruskal–Wallis test followed by Dunn’s multiple-comparison post hoc test with Benjamini–Hochberg *p*-value adjustment. Data are presented as box-and-whisker plots overlaid with individual data points on a logarithmic scale. Governorates sharing the same letter are not significantly different, whereas different letters indicate significant differences (*p* < 0.05).

ELISA antibody titers differed significantly among governorates (*p* < 0.05; Figure 2). Higher antibody titers were observed in Beheira, Dakahlia, Fayoum, Gharbia, Giza, Ismailia, Kafr El-Sheikh, and Sharkia, which clustered within the same statistical group (“a”). In contrast, significantly lower titers were detected in Assiut, Minya, and Port Said (group “b”). Although variability in antibody levels was observed within several governorates, the overall distribution demonstrated clear spatial differences in serological responses among the surveyed governorates.

**Figure 3:**
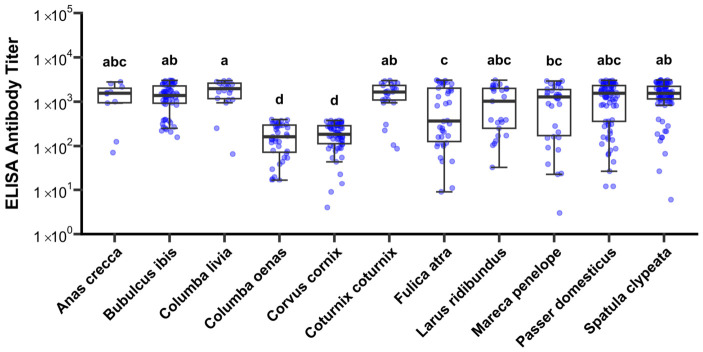
Species-specific ELISA antibody titers against avian metapneumovirus (aMPV) in wild birds across Egypt (2024–2026). Differences were assessed using the Kruskal–Wallis test, followed by pairwise post hoc comparisons with Dunn’s test and *p*-value adjustment according to Benjamini–Hochberg. Data are presented as boxplots with individual data points. Species that do not share the same letter differ significantly (*p* < 0.05).

Significant differences in antibody titers were also observed among species (*p* < 0.05; Figure 3). The highest responses were detected in *Anas crecca*, *Bubulcus ibis*, *Columba livia* and *Coturnix coturnix*, *Larus ridibundus*, *Passer domesticus*, and *Spatula clypeata*. Moreover, *Columba livia* showed higher antibody titers compared to *Mareca penelope*, while *Fulica atra* showed lower antibody titers compared to *Bubulcus ibis*, *Columba livia* and *Coturnix coturnix*, and *Spatula clypeata*. Finally, the lowest antibody titers were observed in *Columba oenas* and *Corvus cornix*.

**Figure 4:**
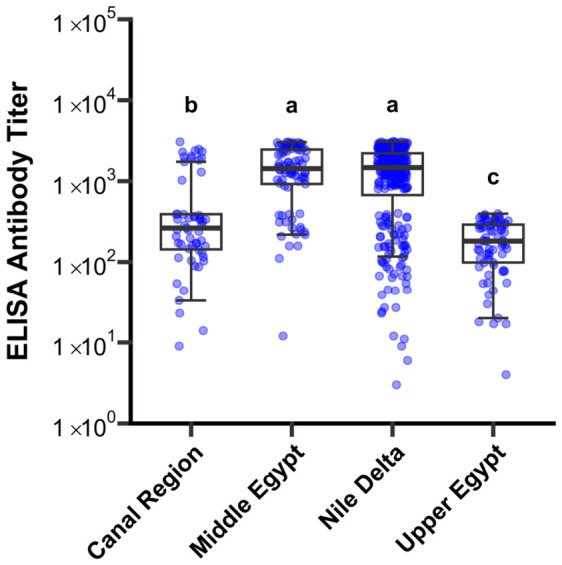
Regional distribution of ELISA antibody titers against avian metapneumovirus (aMPV) in wild birds sampled across Egypt during 2024–2026. Differences among regions were evaluated using the Kruskal–Wallis test followed by Dunn’s multiple-comparison post hoc test with Benjamini–Hochberg *p*-value adjustment. Data are presented as box-and-whisker plots overlaid with individual data points on a logarithmic scale. Regions sharing the same letter are not significantly different, whereas different letters indicate significant differences (*p* < 0.05).

ELISA antibody titers differed significantly among geographic regions (*p* < 0.05; Figure 4). The highest antibody titers were observed in the Nile Delta and Middle Egypt regions, which clustered within the same statistical group (“a”). Intermediate titers were detected in the Canal Region (group “b”), whereas significantly lower titers were recorded in Upper Egypt (group “c”). Although substantial variability in antibody levels was observed within some regions, particularly the Nile Delta, the overall distribution demonstrated clear regional differences in serological responses among the surveyed wild bird populations.

#### 3.2.2. Assessment of ELISA Reactivity Specificity

ELISA-positive sera obtained from Passer domesticus, Spatula clypeata, and Bubulcus ibis demonstrated progressive reduction in ELISA OD450 values following pre-incubation with homologous aMPV subtype B antigen (Supplementary Table S2). The highest inhibition percentages were observed at lower antigen dilutions (1:2), reaching 69.0%, 74.8%, and 61.2% for Passer domesticus, Spatula clypeata, and Bubulcus ibis, respectively.

Inhibition progressively decreased with increasing antigen dilution, consistent with reduced competitive blocking at lower antigen concentrations.

**Figure 5:**
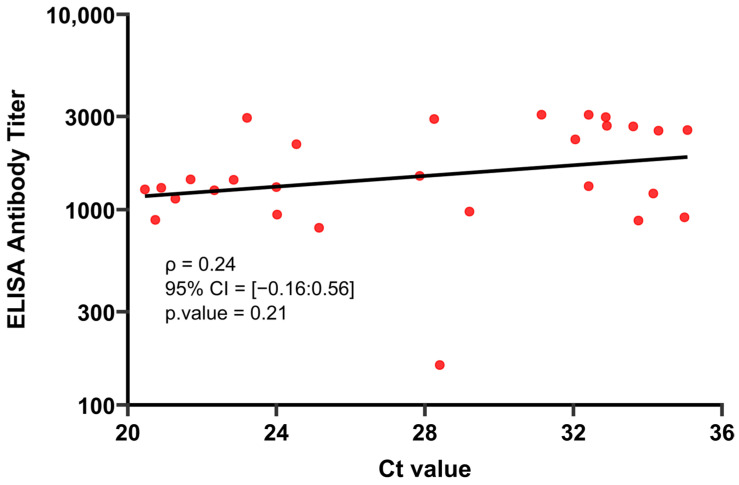
Relationship between RT-qPCR Ct values and corresponding ELISA antibody titers against avian metapneumovirus (aMPV) in PCR-positive wild birds sampled across Egypt during 2024–2026 (*n* = 28). The association was evaluated using Spearman’s rank correlation analysis. The solid line represents the fitted trend line while the red dots represent individual data points. The calculated Spearman correlation coefficient (*ρ*), 95% confidence interval, and *p*-value are shown within the figure.

No statistically significant correlation was identified between ELISA antibody titers and RT-qPCR Ct values among PCR-positive wild bird samples (Spearman’s *ρ* = 0.24, *p* = 0.21; Figure 5). Although a weak positive trend was observed, antibody titers showed substantial variability across the Ct value range, and high or low antibody titers were detected at both lower and higher Ct values. These findings indicate the absence of a consistent monotonic relationship between viral RNA detection levels and corresponding serological responses in the sampled wild birds.

## 4. Discussion

Understanding respiratory virus circulation at the wildlife–poultry interface remains a central challenge in avian disease ecology, particularly in regions where major migratory flyways intersect with intensive poultry production systems. In the present study, integrated molecular and serological surveillance revealed widespread serological reactivity across ecological groups, contrasted by a relatively low molecular detection rate (3.5%). Notably, this discrepancy is biologically consistent with the infection dynamics of aMPV, in which viral shedding is transient and restricted to a short post-infection window, whereas antibody responses persist for considerably longer periods [31,32,33,39]. Accordingly, the findings are more indicative of cumulative exposure events within interconnected avian populations than of active or sustained viral replication in wild birds.

At the molecular level, all detected viruses were identified as subtype B, while subtype A was not detected in any sampled birds. This finding is consistent with previous reports describing the dominance of subtype B in Egyptian poultry systems and its repeated detection in wild birds associated with poultry-linked environments [18,19,20]. Similarly, subtype B predominance has been reported in Europe, North and South America, where detections in wild birds are frequently associated with intensive poultry production areas [40,41]. Therefore, the exclusive detection of subtype B in the present study should be interpreted within a broader epidemiological and ecological context rather than as an isolated observation.

Importantly, this subtype distribution must be interpreted in light of the structural complexity of the Egyptian poultry industry. Poultry production systems are highly heterogeneous with respect to management practices, vaccination strategies, and biosecurity implementation. On the one hand, commercial breeder and layer operations generally implement structured vaccination programs against aMPV, including both live attenuated and inactivated vaccines. On the other hand, broiler flocks are frequently inconsistently vaccinated or remain unvaccinated due to their short production cycle and economic constraints. In addition, variability in biosecurity implementation, particularly in small-scale and backyard systems, may facilitate environmental viral persistence and inter-farm transmission [42,43]. Under these conditions, viral circulation may continue despite vaccination due to incomplete coverage, suboptimal administration, waning immunity, environmental contamination, or antigenic divergence between field and vaccine strains. Collectively, these factors create a complex epidemiological network in which poultry–environment interfaces may function as repeated zones of viral exposure for wild birds.

Regarding serology, exposure was detected across both synanthropic and migratory species, indicating that aMPV-associated antigen exposure occurs in multiple ecological compartments. Synanthropic species such as *Passer domesticus* and *Columba livia* exhibited relatively high seroprevalence despite limited molecular detection, which may reflect repeated environmental exposure to peri-domestic habitats. Consistent with this, previous studies have shown that these species may seroconvert following exposure without sustaining prolonged viral replication or efficient transmission [44,45]. However, the present cross-sectional design does not allow definitive inference regarding their epidemiological role.

Similarly, migratory waterfowl including Spatula clypeata, Mareca penelope, and Anas crecca showed measurable serological exposure with low molecular detection rates. Comparable patterns have been reported in Europe and North America, where wild aquatic birds are generally considered transiently exposed hosts rather than long-term reservoirs [7,8,9,46,47]. Thus, the present findings are consistent with intermittent exposure at shared wetland ecosystems; however, they do not support inference of directional transmission or long-distance dissemination without longitudinal or phylogenetic evidence.

From a spatial perspective, both molecular and serological results demonstrated apparent ecological structuring. Higher exposure levels were observed in the Nile Delta and Middle Egypt, intermediate levels in the Canal Region, and absence of detectable infection or seropositivity in Upper Egypt. This gradient likely reflects differences in poultry density, wetland distribution, migratory bird congregation sites, and intensity of wildlife–poultry interaction. Notably, the Nile Delta and Canal regions represent major ecological convergence zones where dense poultry production overlaps with wetlands and aquaculture systems, thereby increasing opportunities for indirect viral exposure [20,43,48]. Similar spatial clustering has been documented for avian respiratory viruses in other wetland-associated production systems worldwide [49,50,51,52].

Although significant differences in antibody levels were observed among species and governorates, these findings should be interpreted with caution because species distribution was not fully independent of geographic location in some instances. In particular, certain species were sampled exclusively within a single governorate, resulting in partial confounding between species and spatial effects. For example, Columba oenas was sampled only in Minya, and thus any observed serological pattern for this species cannot be disentangled from governorate-level effects. Under such circumstances, it is not possible to determine whether the observed variation in seroprevalence reflects a true geographic effect, a species-specific effect, or a combination of both. Therefore, the apparent spatial heterogeneity may partially reflect the nested and uneven structure of the sampling design rather than fully independent ecological drivers.

In terms of integration between datasets, no statistically significant correlation was observed between ELISA antibody titers and RT-qPCR Ct values among molecularly positive birds. Although a weak positive trend was noted, antibody levels varied considerably across the Ct range. This is expected biologically, as Ct values reflect viral RNA load at a single time point, whereas antibody titers represent cumulative or past exposure over variable infection timelines.

Methodologically, interpretation of serological signals requires careful consideration of host-dependent variability in wildlife systems. The ELISA platform used in this study was originally optimized for domestic poultry and may therefore be influenced by interspecies differences in immunoglobulin structure, binding affinity, and secondary antibody interactions across phylogenetically diverse wild birds [53,54].

To address this limitation, a supplementary inhibition assay demonstrated antigen-dependent reduction in ELISA reactivity following pre-incubation with homologous aMPV subtype B antigen. This strongly suggests specific antibody–antigen interaction and reduces the likelihood that observed signals are due to nonspecific background binding. Collectively, these findings support the biological relevance of the serological data while acknowledging inherent limitations of cross-species assay application.

Taken together, the present study provides integrated molecular and serological evidence supporting circulation of aMPV subtype B in wild birds inhabiting ecologically connected poultry-associated environments in Egypt. However, several limitations must be considered. The cross-sectional design limits temporal inference and prevents determination of transmission directionality. Sampling heterogeneity across species and regions may have influenced prevalence estimates. Most importantly, the absence of viral sequencing precludes differentiation between field, vaccine-derived, or recombinant strains, which is particularly relevant in settings where live attenuated subtype B vaccines are widely used.

Finally, future studies should incorporate targeted RT-PCR sequencing of the G gene and/or whole-genome approaches to improve molecular resolution. In addition, longitudinal multi-season surveillance and integrated poultry–wildlife comparative frameworks will be essential to clarify transmission dynamics and define the epidemiological role of wild birds within the broader aMPV ecology in Egypt.

## 5. Conclusions

The present study demonstrated molecular and serological evidence of avian metapneumovirus subtype B exposure among wild birds inhabiting poultry-associated ecological interfaces in Egypt. Molecular detection rates were relatively low, whereas serological exposure was widespread, supporting a pattern consistent with transient infection and broader cumulative exposure within ecologically connected avian populations.

These findings provide baseline surveillance data for aMPV circulation at the wildlife–poultry interface in Egypt and highlight the importance of integrated molecular and serological monitoring in ecologically complex poultry production systems. Future studies incorporating longitudinal surveillance, phylogenetic analysis, and broader ecological sampling will be essential to clarify transmission dynamics and distinguish circulating field strains from potential vaccine-associated viruses.

## Figures and Tables

**Table 1:** Distribution of molecular and serological detection of avian metapneumovirus (aMPV) in wild birds across Egyptian governorates and ecological regions.

Region	Governorate	Species (Scientific Name)	Common Name	Ecological Behaviour	No. Tested (PCR)	No. Positive	Prevalence (%)	No. Tested (ELISA)	No. Positive	Seroprevalence (%)
Nile Delta	Beheira	*Passer domesticus*	House sparrow	Synanthropic, granivorous	40	3 ^B^	7.5	20	13	65
		*Spatula clypeata*	Northern shoveler	Migratory dabbling duck	55	6 ^B^	10.91	55	44	80
		*Mareca penelope*	Eurasian wigeon	Migratory grazing duck	30	2 ^B^	6.67	15	15	100
		*Anas crecca*	Green-winged teal	dabbling duck	10	0	0.0	10	8	80
	Kafr El-Sheikh	*Spatula clypeata*	Northern shoveler	Migratory dabbling duck	72	5 ^B^	6.9	30	29	96.67
		*Mareca penelope*	Eurasian wigeon	Migratory grazing duck	4	1 ^B^	25 *	4	4	100
		*Fulica atra*	Eurasian coot	wetland	80	1 ^B^	1.25	18	2	11.11
	Sharkia	*Mareca penelope*	Eurasian wigeon	Migratory grazing duck	20	1 ^B^	5.0	15	4	26.6
		*Columba livia*	Rock Dove	Synanthropic terrestrial granivorous	20	1 ^B^	5.0	20	18	90
	Dakahlia	*Fulica atra*	Eurasian coot	Wetland-associated rail	40	1 ^B^	2.5	20	16	80
		*Coturnix coturnix*	Common quail	Ground-dwelling migratory granivorous	30	1 ^B^	3.3	25	21	84
	Gharbia	*Passer domesticus*	House sparrow	Synanthropic, granivorous	62	1 ^B^	1.6	40	30	75
Middle Egypt	Fayoum	*Bubulcus ibis*	Cattle egret	Livestock-associated insectivore	92	0	0.0	43	37	86
	Giza	*Passer domesticus*	House sparrow	Synanthropic, granivorous	20	1 ^B^	5.0	20	15	75
		*Bubulcus ibis*	Cattle egret	Livestock-associated insectivore	52	1 ^B^	1.9	18	11	61
Canal Region	Ismailia	*Larus ridibundus*	Black-headed gull	Migratory wetland-associated omnivorous	30	2 ^B^	6.7	25	13	52
	Port Said	*Corvus cornix*	Hooded crow	Terrestrial omnivore	42	1 ^B^	2.4	30	0	0.0
Upper Egypt	Minya	*Columba oenas*	Stock dove	Synanthropic/peri-domestic	61	0	0.0	39	0	0.0
	Assiut	*Corvus cornix*	Hooded crow	Terrestrial omnivore	40	0	0.0	33	0	0.0
Total	—	—	—	—	800	28	3.5	480	280	58.3

* Some Pervalence Percentages were calculated from small sample sizes. ^B^ refers to aMPV subtype B.

## Data Availability

The datasets generated and/or analyzed in the current study are included in the manuscript. Additional data is available from the corresponding author upon reasonable request.

## Supplementary Materials

The following supporting information can be downloaded at: https://www.mdpi.com/article/10.3390/v18060591/s1, Supplementary Figure S1. Distribution of ELISA antibody titers for all analyzed serum samples, the histogram represents the observed frequency of ELISA antibody titers, and the overlaid curve depicts the kernel density of estimation of the distribution. The manufacturer-provided (kit) cutoff and calculated cutoff derived from density valley analysis are indicated by dashed vertical lines. Supplementary Table S1. Raw metadata for wild birds sampled during surveillance in Egypt, including geographic origin, species, RT-qPCR result (1 = positive, 0 = negative) with Ct values, and ELISA titer with serological classification (1 = positive, 0 = negative). Samples without ELISA data were not serologically tested. Supplementary Table S2. Antigen inhibition assay demonstrating reduction in ELISA reactivity in selected wild bird sera following pre-incubation with vaccinal avian metapneumovirus (aMPV) subtype B antigen. Progressive reduction in OD450 values and corresponding inhibition percentages supported the specificity of the detected serological reactivity. Negative inhibition values at higher antigen dilutions likely reflected reduced competitive blocking activity.

## Abbreviations

Abbreviations are defined in the text upon first mention and are listed here alphabetically:

Abbreviation	Full Term
aMPV	Avian metapneumovirus
aMPV-A	Avian metapneumovirus subtype A
aMPV-B	Avian metapneumovirus subtype B
Ct	Cycle threshold
ELISA	Enzyme-linked immunosorbent assay
FAM	6-carboxyfluorescein (fluorescent reporter dye)
FBS	Fetal bovine serum
HBSS	Hanks’ Balanced Salt Solution
HRP	Horseradish peroxidase
IACUC	Institutional Animal Care and Use Committee
IDVET	IDvet diagnostic kit (commercial ELISA platform)
Kylt	Kylt^®^ molecular diagnostic kit
OD	Optical density
ODNC	Optical Density of negative control
ODPC	Optical Density of positive control
PCR	Polymerase chain reaction
RNA	Ribonucleic acid
RT-qPCR	Real-time reverse transcription polymerase chain reaction
S/P	Sample-to-positive ratio
TMB	Tetramethylbenzidine
VTM	Viral transport medium
