# Supplementary material for: Avian Metapneumovirus Subtype B at the Wildlife–Poultry Interface in Egypt: Molecular and Serological Insights into Cross-Ecological Transmission
Source: Viruses. 2026 May 24;18(6):591. doi: 10.3390/v18060591 (PMC13308320; doi:10.3390/v18060591)
Supplement: Supplementary file 1 [file viruses-18-00591-s001.zip › Quantitative Inhibiton Assesment of ELISA Supplementary Table 2.pdf]

Supplementary Table 2. Antigen inhibition assay demonstrating reduction of ELISA reactivity in selected wild bird sera following pre-incubation with vaccinal avian metapneumovirus (aMPV) subtype B antigen. Progressive reduction in OD450 values and corresponding inhibition percentages supported the specificity of the detected serological reactivity. Negative inhibition values at higher antigen dilutions likely reflected reduced competitive blocking activity.

Sample ID	Species	Antigen Dilution	Serum Test OD450	Inhibition (%)
**BEH_PA_001**	*Passer domesticus*	none	1.32	0.0
		1:2	0.409	69.0
		1:4	0.558	57.7
		1:8	0.744	43.6
		1:16	0.93	29.5
		1:32	1.116	15.5
**BEH_SP_001**	*Spatula clypeata*	none	1.65	0.0
		1:2	0.416	74.8
		1:4	0.567	65.6
		1:8	0.756	54.2
		1:16	0.945	42.7
		1:32	1.134	31.3
**GIZ_BU_001**	*Bubulcus ibis*	none	1.01	0.0
		1:2	0.392	61.2
		1:4	0.534	47.1
		1:8	0.712	29.5
		1:16	0.89	11.9
		1:32	1.068	-5.7
