# Supplementary material for: Avian Metapneumovirus Subtype B at the Wildlife–Poultry Interface in Egypt: Molecular and Serological Insights into Cross-Ecological Transmission
Source: Viruses. 2026 May 24;18(6):591. doi: 10.3390/v18060591 (PMC13308320; doi:10.3390/v18060591)
Supplement: Supplementary file 1 [file viruses-18-00591-s001.zip › Supplementary figure S1.pdf]

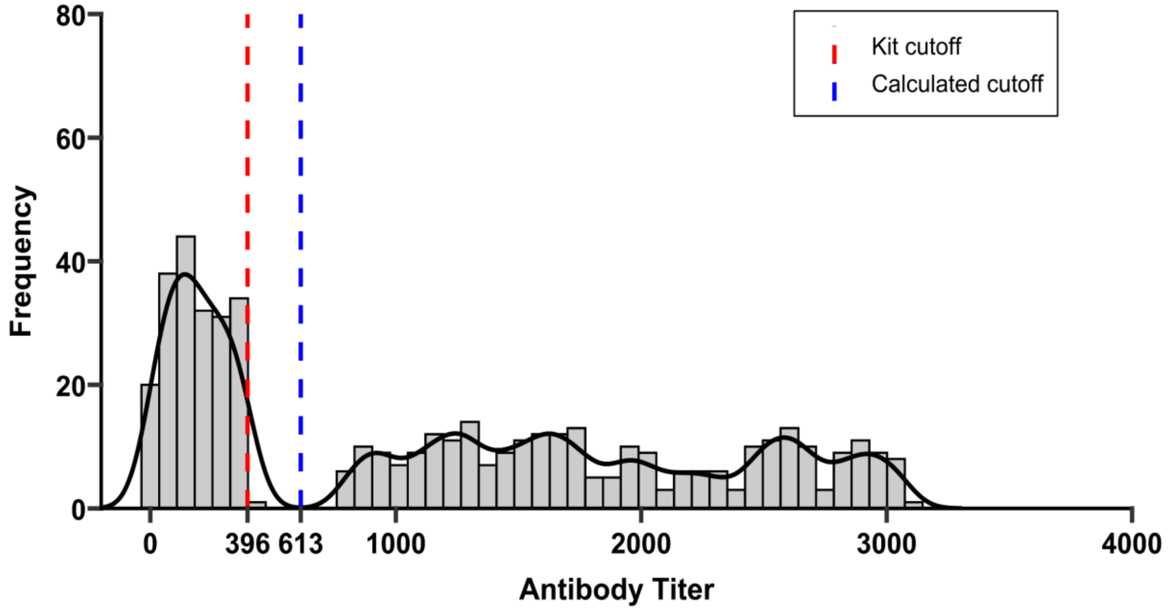

Supplementary Figure 1. Distribution of ELISA antibody titers for all analyzed serum samples. The histogram represents the observed frequency of ELISA antibody titers, and the overlaid curve depicts the kernel density estimation of the distribution. The manufacturer-provided (kit) cutoff and the calculated cutoff derived from density valley analysis are indicated by dashed vertical lines.
